# UV Light Degradation of Polylactic Acid Kickstarts Enzymatic Hydrolysis

**DOI:** 10.1021/acsmaterialsau.3c00065

**Published:** 2023-11-02

**Authors:** Margaret H. Brown, Thomas D. Badzinski, Elizabeth Pardoe, Molly Ehlebracht, Melissa A. Maurer-Jones

**Affiliations:** Department of Chemistry and Biochemistry, University of Minnesota Duluth, 1038 University Dr, Duluth, Minnesota 55812, United States

**Keywords:** polylactic acid, biodegradability, abiotic degradation, UV light, enzymatic hydrolysis

## Abstract

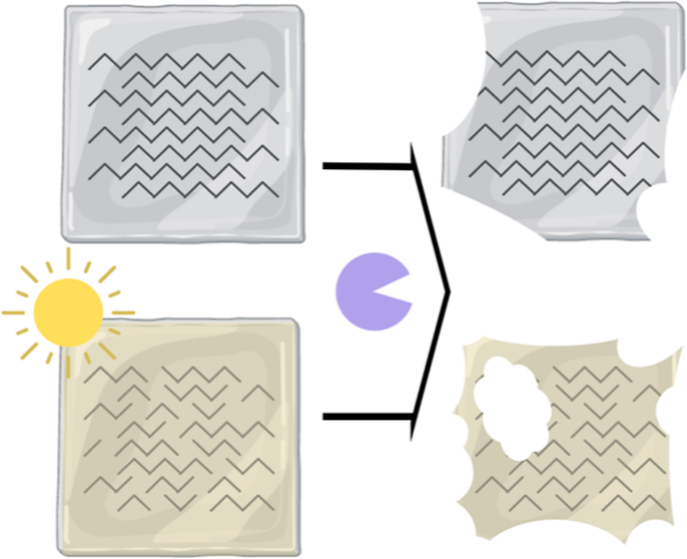

Polylactic acid (PLA) and bioplastics alike have a designed degradability to avoid the environmental buildup that petroplastics have created. Yet, this designed biotic-degradation has typically been characterized in ideal conditions. This study seeks to relate the abiotic to the biotic degradation of PLA to accurately represent the degradation pathways bioplastics will encounter, supposing their improper disposal in the environment. Enzymatic hydrolysis was used to study the biodegradation of PLA with varying stages of photoaging. Utilizing a fluorescent tag to follow enzyme hydrolysis, it was determined that increasing the amount of irradiation yielded greater amounts of total enzymatic hydrolysis by proteinase K after 8 h of enzyme incubation. While photoaging of the polymers causes minimal changes in chemistry and increasing amounts of crystallinity, the trends in biotic degradation appear to primarily be driven by photoinduced reduction in molecular weight. The relationship between photoaging and enzyme hydrolysis appears to be independent of enzyme type, though commercial product degradation may be impacted by the presence of additives. Overall, this work reveals the importance of characterizing biodegradation with relevant samples that ultimately can inform optimization of production and disposal.

## Introduction

Given the critical role plastics play in daily life, biodegradable polymers seek to combat the environmental problems that have been caused through mismanagement of petro-plastic waste. Bioplastics like polylactic acid (PLA) offer a promising alternative to petroplastics because it is expected that their designed biodegradability will give another pathway of breakdown and disposal that is not afforded to the legacy polymer products. The popularity of such innovative polymers continues to grow, with a projected production rate of over 6.3 million tons in 2027. Of these 6.3 million tons, 37.9% of production is expected to be PLA and have a market value of over 2.2 billion USD.^1−3^ As the Bioplastic market expands, it becomes imperative to ensure these new materials do not add to current pollution problems.

Should they enter the environment unintentionally, PLA weathers through chemical and physical processes. Photodegradation is noted as the most common abiotic degradation driving force.^4^ In PLA, the effects of photodegradation include an increase in brittleness, crystallinity, hydrophilicity, and a reduction of molecular weight.^5^ Similar changes and trends have been observed in petroplastics.^6−8^ When applied to the biodegradation of petroplastics, the relative crystallinity drives the polymer’s ability to biodegrade, that is, crystallinity prevents the adsorption of microorganisms to polymer surfaces, reducing the biodegradation capabilities.^9−11^ Thus, the weathering induced changes in properties greatly affect the polymer’s ability to degrade.

Currently, much of the work characterizing the biodegradability of these new materials occur under the most ideal conditions,^9,12−17^ and do not typically account for changes to the polymer throughout its use and disposal. The designed degradability of biodegradable plastics can be delayed or altered by the makeup of commercial products (i.e., the use of additives) or its state of degradation.^18−21^ Ultimately, this could cause more waste accumulation in the environment for which mitigation techniques are currently no different from those for petroplastic waste. Therefore, it is critical to consider end-of-life conditions that plastic products experience, including additives and the state of weathering of the plastics that could prevent wide scale commercial integration of bioplastics.

Enzyme hydrolysis provides a tool to investigate the effects polymer weathering has on the designed biodegradability of PLA. As the first step in total biodegradation of plastics, enzymatic hydrolysis results in the cleavage of polymers chains into monomers, dimers, and oligomers capable of being mineralized by microorganisms.^22,23^ Given the importance of enzymatic hydrolysis for total biodegradation, it is the process that this study tracks to evaluate biotic degradation. Further, measuring enzymatic hydrolysis is the prevailing approach to evaluate biodegradability within the literature.^9,20,21,24−27^ However, there exists a gap within current knowledge of environmental degradation that encapsulates both abiotic and biotic factors. Herein, this study demonstrates the relationship between photo- and biodegradation in PLA and reveals that rather than crystallinity hindering biodegradation, decreases in polymer molecular weight boost biodegradation with photoaging. Ultimately, biodegradation measurements indicate that photoweathering promotes the enzymatic hydrolysis in aged PLA samples.

## Materials and Methods

### Plastic and Plastic Preparation

Poly-*l*-lactic acid (PLLA) thin film samples used in this study were 50 μm thick (Goodfellow; Huntingdon, GB). PLLA granules of different molecular weights were purchased from Polysciences (Warington, PA). To study the effects of additives, we used commercially available plastic cutlery (World Centric) and common commercial additives were used. In experimentation, plastic cutlery was used as-is. Additive doped polymers were pristine PLLA samples spiked with commercially relevant amounts of titanium dioxide (TiO_2_; 4% w/w; a coloring agent) (CAS#13463-67-7), Bis (2,2,6,6-tetramethyl-4-piperidyl) (3% w/w; a UV stabilizer) (CAS#52829) or talc (11% w/w; a bulking agent) (CAS#14807-96-6). Prior to experiments, polymer thin film samples were soaked for 24 h in each of hexanes (CAS#110-54-3), followed by methanol (CAS#67-56-1), and then doubly distilled water to remove unpolymerized monomers/oligomers or processing additives. This leaching process was not employed for cutlery or molecular weight standards.

Accelerated UV degradation was performed in a Rayonet merry-go-round photochemical reactor (Southern New England Ultraviolet Co.; Branford, CT) by irradiating films on both sides for various time periods with 16 Hg-vapor lamps (SNE Ultraviolet Co RMR-2537A; Bamford, CT) emitting photons centered at 300 nm light. PLLA was aged for 0, 1, 2, 3, 4, 8, and 24 h per side in this accelerated artificial fashion to produce equivalent natural aging byproducts.^8^

### Enzymatic Hydrolysis Tracking with Fluorescence Spectroscopy

To quantitatively track the enzymatic hydrolysis of aged PLLA samples, a fluorogenic-labeling method was used based on a previous study.^28^ Briefly, samples were prepared in a chloroform solution with PLLA [2% (w/w)] and fluorescein dilaurate (0.002% w/w FDL; (CAS#7308-90-9) (Sigma-Aldrich)). Samples with additives were cast with the same PLLA and FDL concentration, keeping the amount of PLLA consistent in the cast between the different additives. Samples were cast into the bottom of 10 mm Starna Cells quartz cuvettes (Atascadero, CA) and allowed to dry completely for a minimum of 12 h. This results in approximately a 30 μm thick film at the bottom of the cuvette. Samples were prepared in quadruplicate to produce triplicate enzymatic hydrolysis measurements, along with a reference. Prior to casting the doped-polymer solutions, cuvettes were cleaned in Hellmanex III (Z805939) to ensure that protein did not bind to the glass surface during experimentation.

Proteinase K (CAS# 39450-01-6) was purchased from Research Products International (Mt. Prospect, IL) and used at a concentration of 30 μM in Tris HCl (CAS# 1185-53-1) at pH 7.5 for all samples. The concentration of enzyme was chosen based on experimentation to optimize enzyme binding as described in the Supporting Information (Figure S1). Experiments were run in batches at 37 °C. The protein solution was added to the cuvette immediately before the start of fluorescence measurements.

Fluorescence measurements were taken on a Horiba Fluoromax-4 Spectrofluorometer (Horiba Scientific; Edison, NJ) with an attached Quantum Northwest Temperature Control Turret with 4 cuvette slots (Liberty Lake, WA) and Koolance EXT-440 Liquid Cooling System (Auburn, WA). Excitation was 485 nm, and emission spectra were collected from 500 to 650 nm with a 4 nm slit width for both excitation and emission. Measurements were taken in triplicate every 5 min over a span of 8 h. An additional reference cuvette with doped-PLLA with only Tris HCL buffer was run concurrently with the enzyme samples.

To analyze the fluorescence data, the reference cuvette signal was subtracted from each sample cuvette to account for any autohydrolysis and release of the fluorogenic probe (see Supporting Information Figures S6 and S7). At lower levels of hydrolysis, the fluorescence signal at 511 nm was used to convert into a concentration of fluorescein using a fluorescein calibration curve (Supporting Information Figure S5). When high concentrations of fluorescein were released, a bathochromic spectral shift was observed, and calibration of the concentration of fluorescein was calibrated to the peak wavelength value (see Supporting Information Figures S3 and S4 for more information). The fluorescein concentration was converted into percent (%) ester bonds by comparing the amount of fluorescein, which was formed when 2 ester bonds per FDL molecule were cleaved, to the number of ester bonds within casted films. Ultimately, the values were converted into the amount of ester bonds broken in PLLA and percent ester linkages broken by comparing to the total number of ester bonds in the cast PLLA. It should be noted that switching the calibration methods may have induced artifacts in the observed hydrolysis rates. However, when comparing total ester bonds broken values between different polymer samples, we ensured the fluorescein concentrations, and thus the ester cleavage percent, were calculated with the same calibration methods, allowing us to evaluate trends of biodegradation between different sample types.

### Attenuated Total Reflectance—Fourier Transform Infrared Spectroscopy

Attenuated total reflectance—Fourier transform infrared (ATR–FTIR) spectroscopy was performed on the samples to characterize the molecular structures on the polymer surface. A Nicolet iS50 (Thermo Fisher Scientific; Waltham, MA) with a diamond ATR cell was used. Spectra were collected at a minimum of 3 locations per sample. Each spectrum was an average 64 scans with a 4 cm^–1^ resolution. Analysis of the carbonyl and hydroxyl bands in the spectra is a measure of the degree of weathering, as both are well established to grow with photodegradation.^4,11^ To quantify the carbonyl band, ATR–FTIR measurements of aged PLLA samples were analyzed using Igor Pro 8.04 (WaveMetrics; Portland, OR) software, individually analyzing the spectra of carbonyl absorption band area (1730–1800 cm^–1^) and normalized to a reference band area (∼1455 cm^–1^). This ratio generated a single value known as a carbonyl index. Similarly, a hydroxyl index was calculated using the hydroxyl absorption band area (3230–3700 cm^–1^) and the reference band area. The analysis was conducted on each triplicate measurement for plastic samples, and the mean and standard deviation between trials was calculated.

### Differential Scanning Calorimetry

Differential scanning calorimetry (DSC) measurements were conducted using an established method for PLLA on a TA Instruments DSC 250+ calorimeter (New Castle, DE, USA). Samples were analyzed in triplicate using between 5 and 10 mg of the thin film sample in each Tzero pans. Samples were analyzed using a heat–cool–heat cycle, with the temperatures ranging from 25 to 170 °C at a ramp rate of 10 °C/min. Analysis was conducted on the first heating of the samples. Heat flow measurements were automatically normalized by the sample mass on collecting. The enthalpy of melting was used to determine the bulk crystallinity, where enthalpy was normalized to the enthalpy of melting a 100% crystalline sample (93.6 J/g) to calculate the percent crystallinity of the sample.^29^

### Scanning Electron Microscopy

A Hitachi TM3030 Tabletop Plus scanning electron microscope was used to observe the surface of the PLLA upon enzymatic hydrolysis. Samples were mounted with carbon tape and imaged uncoated at a 5 kV accelerating voltage.

## Results and Discussion

### Aging of PLLA Promotes Enzymatic Hydrolysis

**Figure 1:**
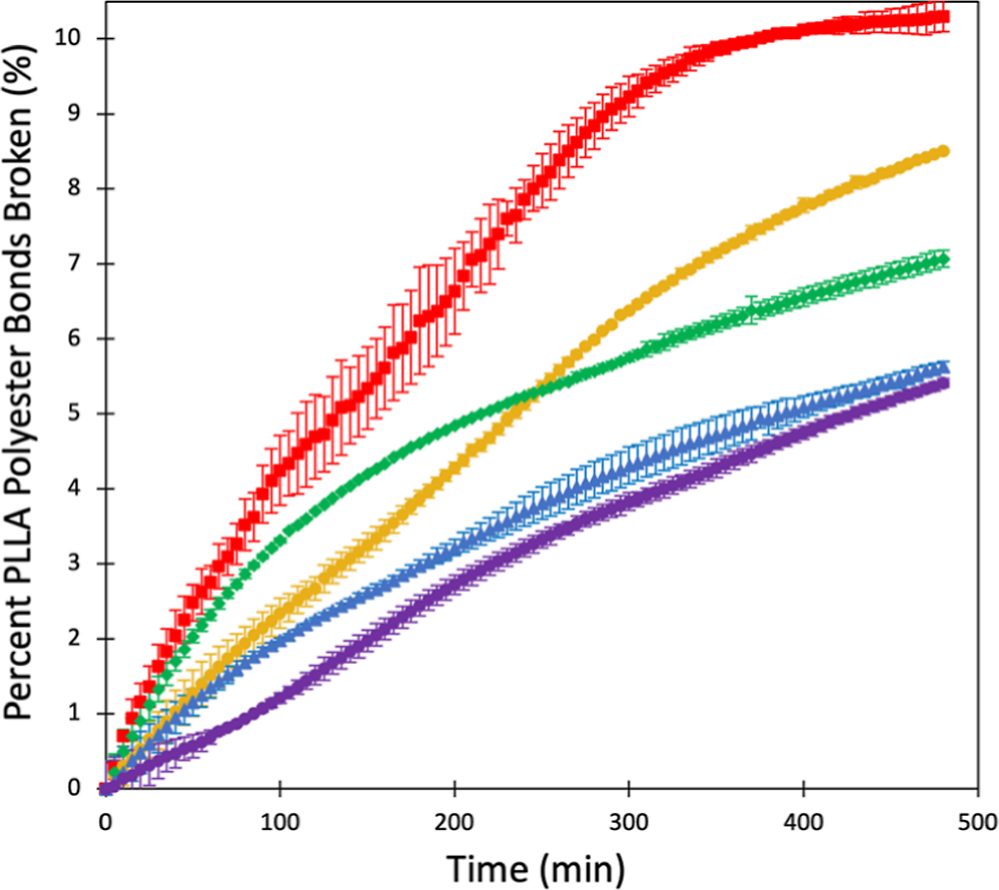
Percentage of PLLA polyester bonds broken over time of pristine (purple), 1 (blue), 2 (green), 3 (gold), and 4 h (red) irradiated PLLA. Markers represent the average of triplicate values with error bars as the standard deviation.

Figure 1 shows the enzymatic hydrolysis, monitored for 8 h, of aged PLLA. As the irradiation time of the plastic increases, the overall number of ester bonds broken over the course of the study increases from 5.4 to 10.3% (Supporting Information Table S1). There appears to be different initial rates where 4 h is fastest and pristine is slowest, but midrange aging does not stay on trend (3 h is slower initially than 2 h). The hydrolysis results maintain the trend where increasing aging causes greater enzymatic breakdown upon 8 h. This is further demonstrated with samples aged for 8 and 24 h (Supporting Information Figure S8 and Table S1), where 24 h of aging reaches up to 80% hydrolysis. We fit the data to reaction rate kinetics (Supporting Information Table S3); however, we also sought to perform experiments to determine if the cause of the changing rates in hydrolysis over the 8 h was the result of changing catalytic behavior of the enzyme (Supporting Information Figures S10 and S11). With a fresh enzyme solution, hydrolysis continued, which indicates that the total values measured in Figure 1 are not the total amount that could be hydrolyzed. To confirm, PLLA samples of unaged and aged (4 h irradiation) were refreshed with enzyme over the course of 3 days. Using mass measurements, it was determined that aged PLLA hydrolyzed more than unaged. Pristine PLLA exhibited a 11.00% mass loss with aged PLLA demonstrating a 14.38% mass loss.

Previous work with PLLA suggests that aging should hinder enzymatic hydrolysis. Cai et al., reported the physical aging of PLLA utilizing thermal degradation hindered total biotic degradation.^9^ These results were correlated to the accompanied increase in crystallinity, a main characteristic change during PLA thermal degradation thought to be caused by a reduction in molecular weight.^9,16,17^ Our work reveals that UV degradation has an opposite effect, where enzymatic hydrolysis is promoted by this aging. This phenomenon has been reported previously by Salač et al., who utilized compost to biodegrade photodegraded PLA.^21^ The study noted that after 90 days, both non- and photodegraded samples reached full mineralization, but that nonirradiated samples lagged at starting stages of biotic degradation.^21^ To understand the transformations driving the observed changes in enzyme hydrolysis, we characterized the material properties.

### Characterizing Aged PLLA Films

**Figure 2:**
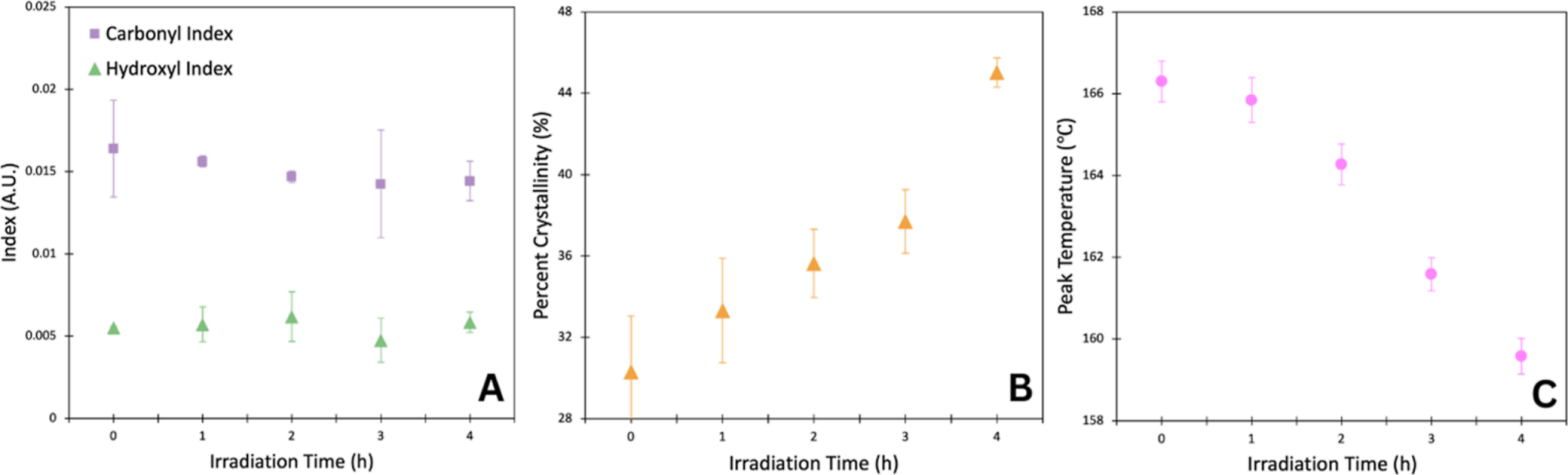
(A) Carbonyl and hydroxyl indices of the irradiated PLLA samples. (B) Percent bulk crystallinity (%) of irradiated PLLA samples. (C) Peak temperature, °C of irradiated PLLA samples from DSC thermograms. Markers represent the average of triplicate values with error bars as the standard deviation.

In order to capture any chemical changes occurring as the result of photoaging, ATR–FTIR analysis of PLLA films were probed to characterize carbonyl and hydroxyl indices. Increases in either index serve as markers for degradation.^4,11^ However, our FTIR analysis proved to be insensitive to significant changes in chemistry, likely because PLA contains oxygen groups intrinsic to its structure that make small changes in these moieties difficult to observe. Figure 2A shows the hydroxyl and carbonyl indices in the polymers upon irradiation. While there appears to be a slight decreasing trend in the carbonyl index, ANOVA tests revealed no statistically significant difference in either index with increased irradiation. This result is not novel; Salač et al., and more recently Campanaro et al., reported no FTIR discernible changes in photodegraded PLLA, respectively.^21,25^ Similarly, carbonyl and hydroxyl indices were quantified following enzyme exposure and again no statistically significant changes were observed with photoaging, though the enzyme caused differences from the nonenzyme exposed samples (Supporting Information Figure S12).

We further investigated the changes in crystallinity to understand its role in total decomposition or potentially its impact on the initial rates of irradiated samples. Bulk crystallinity measurements as determined from the DSC data (Supporting Information Figure S13) revealed an increase in crystallinity with greater levels of aging (Figure 2B). It has been readily determined that highly crystalline polymers, specifically PLLA, inhibit biotic degradation.^9,26,27^ Since the observed trend in our photodegraded samples has increasing crystallinity, this indicates that the susceptibility of UV light degraded PLA to enzymatic hydrolysis must be influenced by a different material property (e.g., density, molecular weight, and tensile strength).

Peak temperature in the DSC thermograms is an established method for comparison of molecular weights.^30^ Further, it has been readily demonstrated that photodegradation causes a reduction in molecular weight, and separately that lower molecular weight PLLA degrades at a faster rate than their high molecular weight counterparts.^31^Figure 2C illustrates that increasing UV exposure causes a decrease in peak temperature, indicating reduction in molecular weight. Ultimately, these results indicate that changes in molecular weight play an integral role in the enzymatic hydrolysis and aging trends demonstrated above. To confirm that DSC peak temperature measurements could accurately detect changes within molecular weight, PLLA molecular weight standards were analyzed with DSC and revealed decreasing peak temperatures with decreased molecular weight (Supporting Information Figure S14 and Table S4). Taking the materials characterization together, photoaging causes minor changes in the PLLA chemistry and the observed changes in crystallinity should have caused decreased enzyme hydrolysis based on the literature;^9,26,27^ therefore, we concluded that the photochemically induced decrease in molecular weight is the primary transformation dictating the efficiency of the enzymatic hydrolysis of aged PLLA.

**Figure 3:**
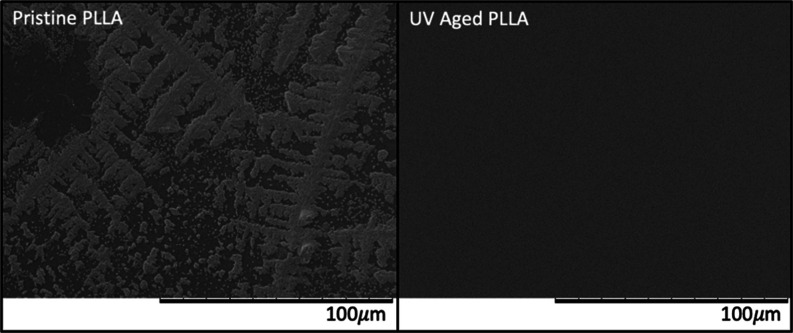
SEM imaging of pristine and aged (8 h UV) PLLA. These are representative images of *n* = 1 sample per condition with >20 images taken per sample.

Scanning electron microscopy (SEM) was performed to observe any changes happening to the polymer surface upon exposure to enzyme. Pristine PLLA exposed to enzyme revealed a branching pattern, while aged PLLA revealed no distinctive landmarks (Figure 3). Taken together with the hydrolysis and polymer characterization results reported above, we concluded that UV degradation causes lower molecular weight chains more homogeneously on the polymer surface and that this uniformity allows for more areas in which the enzyme can attack aged polymers.

### Enzyme Hydrolysis of Molecular Weight Standards

**Figure 4:**
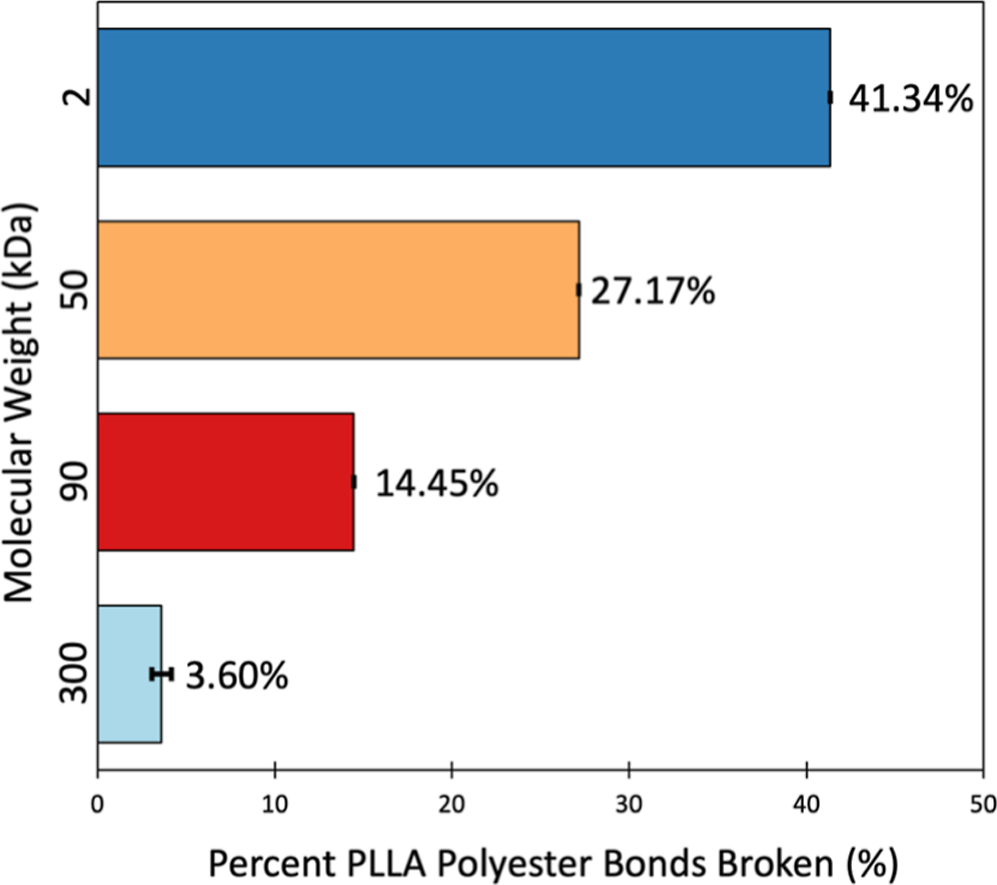
Total percentage of PLLA polyester bonds broken after 8 h of proteinase K exposure in 2, 50, 90, and 300 kDa PLLA molecular weight standards. Values represent the average of 3 replicate samples and error bars as standard deviations.

With DSC observed reduction in molecular weight as a result of aging, we sought to confirm our fluorescence method could accurately track increased enzymatic hydrolysis of lower molecular weight samples, a trend established in the literature.^12^ Our fluorogenic probe was doped into PLLA molecular weight standards (average molecular weights of 2, 50, 90, and 300 kDa; Figure S8). Figure 4 demonstrates that decreasing the molecular weight yields greater amounts of enzymatic hydrolysis over 8 h, confirming that the molecular weight is tied to the ability of PLLA to biodegrade.

### Trends Utilizing Other Enzymes

**Figure 5:**
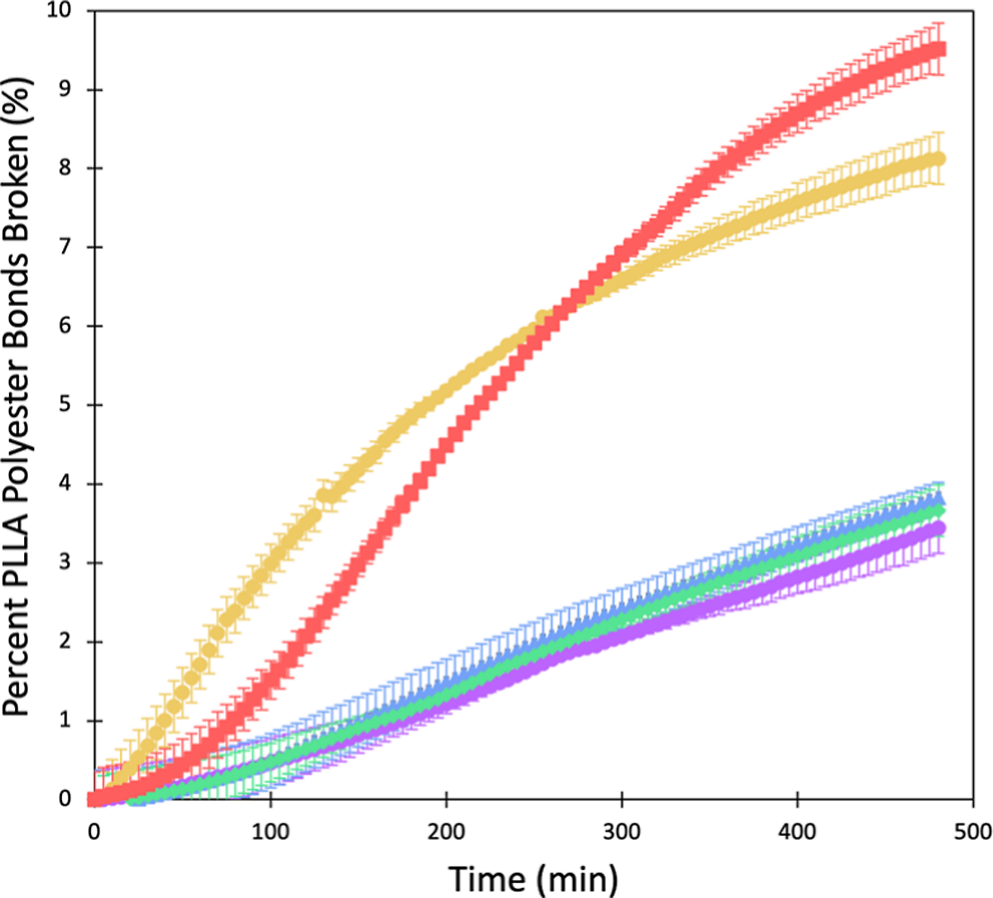
Percentage of PLLA polyester bonds broken over time of pristine (purple), 1 (blue), 2 (green), 3 (gold), and 4 h (red) irradiated PLLA utilizing lipase. Markers represent the average of triplicate values with error bars as the standard deviation.

Our experimentation above used an ideal system for the biotic degradation of PLLA as proteinase K is the leading enzyme for the catalysis of PLLA enzymatic hydrolysis.^32,33^ To determine if the trends demonstrated are more generalizable, the biodegradability experiments were repeated utilizing another esterase, triacylglycerol lipase. Lipases are present in a high majority of living organisms, with a large number of fungi also producing lipases.^34^ Tokiwa et al., demonstrated the ability of lipase to degrade synthetic polyesters, and Alejandra et al., confirmed lipase’s degradation capabilities in poly(3-hydroxybutyrate-*co*-4-hydroxybutyrate).^35,36^ Ultimately, lipase is a readily available enzyme and shown to degrade polyesters.^37,38^Figure 5 shows that lipase induces a trend in enzymatic hydrolysis near identical to that of proteinase K. The presence of the identical catalytic triad in both lipase and proteinase K may be the cause for the similar response, with both proteinase K and lipase exhibit the ‘catalytic triad of aspartic acid (Asp), histidine (His), and serine (Ser).^39^ The catalytic triad catalyzes the nucleophilic attack on carbonyl carbons in esters using the active site serine.^38,40^ While further work exploring wider enzyme structures may yield varied biodegradation capabilities, it is still important that both hydrolases have promoted activity on UV degraded polymers irrespective of individual enzymes.

### Effects of Additives on Enzymatic Hydrolysis

To continue to build on our understanding of the biotic breakdown of relevant polymer samples, such as plastics that are exposed to abiotic factors such as photodegradation, it is also important to study the effects of realistic commercial polymer products that include additives. Initially, to determine if enzyme hydrolysis is impacted by additives, PLA compostable cutlery was studied using the fluorescence methodology. PLA cutlery degraded close to 100% after 4 h, greater than the 12% observed with the 2 kDa PLLA molecular weight standard that had the closest DSC peak melting temperature (158 °C) compared to that of the cutlery (164 °C) (Supporting Information Figure 15).

**Figure 6:**
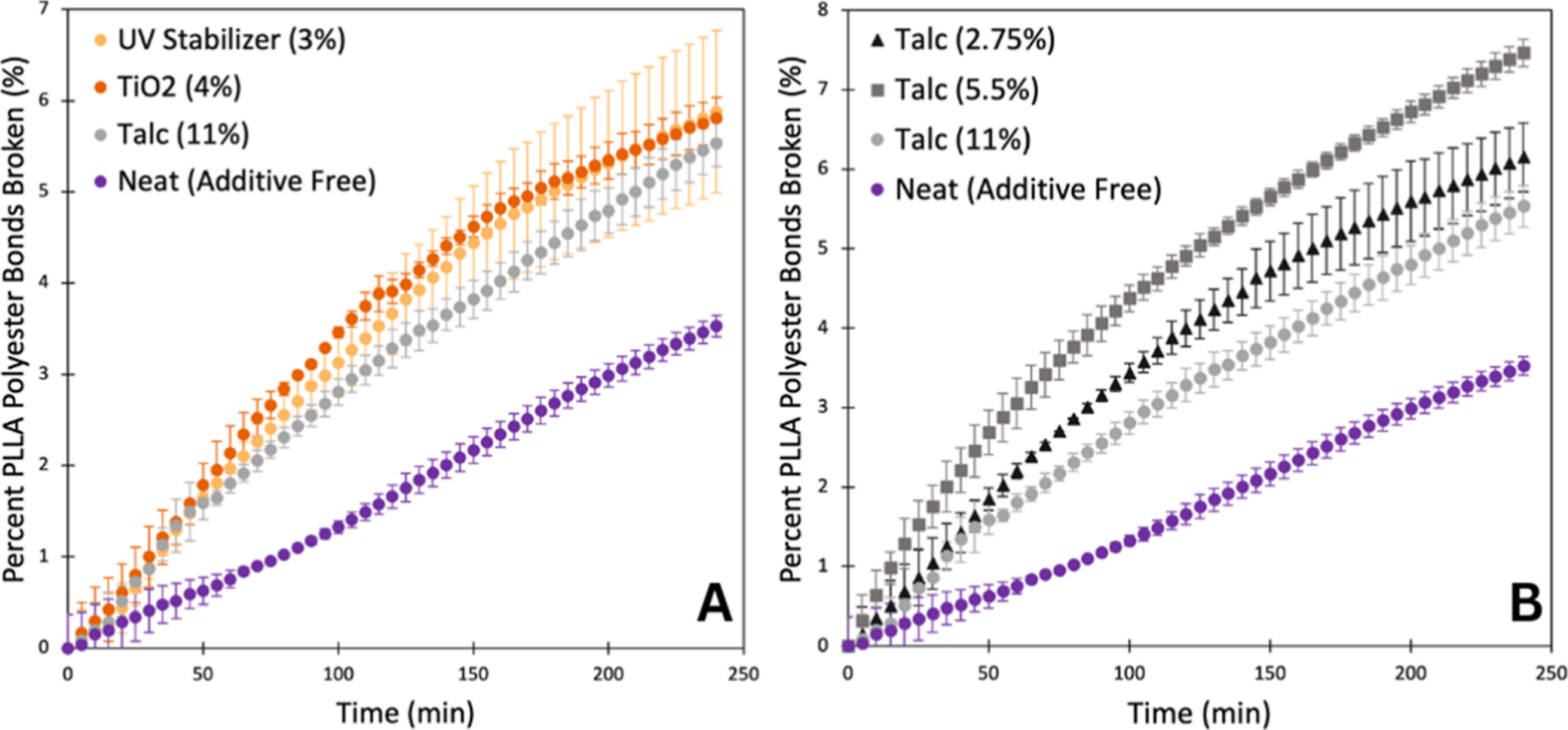
(A) Percentage of PLLA polyester bonds broken over time of pristine PLLA doped with 3% w/w bis (2,2,6,6-tetramethyl-4-piperidyl) (UV Stabilizer), 4% w/w titanium dioxide (TiO_2_), and 11% w/w Talc compared to a neat (additive free) PLLA. (B) Percentage of PLLA polyester bonds broken over time of pristine PLLA doped with 2.75, 5.5, and 11% w/w Talc compared to a neat (additive free) PLLA. Markers represent the average of triplicate values with error bars as the standard deviation.

It was determined gravimetrically that the cutlery was composed of 11% filler, which was reported by the manufacturer to primarily be talc. Further experiments were performed by systematically changing additives that were doped into pristine PLLA (Figure 6A). Similarly, to the PLA cutlery sample, the presence of additives kick-starts enzymatic hydrolysis in the initial stages of degradation. The three additives chosen were titanium dioxide (TiO_2_; a coloring agent) bis [2,2,6,6-tetramethyl-4-piperidyl) a UV stabilizer] and talc (a bulking agent) and were doped at commercially relevant weight percentages. In studies investigating the effects of titanium dioxide (TiO_2_) as an additive in the biodegradation of PLA, it was found to promote the rate of PLA degradation because it increased the ability for water to penetrate the PLA/TiO_2_ mixtures.^41,42^ Conversely for talc, Li and Huneault, reported talc in PLA as a successful nucleating agent, increasing the crystallinity of PLA,^43^ which should decrease biodegradability as others observed in industrial composting conditions.^20,44^ Because our results contradicted previous studies relating the presence of talc to biodegradation, the fluorescent experiments were conducted at varying w/w percentages of talc to PLLA (Figure 6B). As shown, the presence of talc in all weight percentages increased the level of enzymatic hydrolysis over a 4 h period. Interestingly, the middle point of 5.5% (w/w) of talc in PLLA resulted in the greatest total number of polyester bonds broken, indicating that the concentration of additives is ultimately important in the biodegradation of PLLA. However, with a clear bump in degradation from talc presence alone, it is clear that these additives affect PLLA properties, thus boosting enzyme hydrolysis. Overall, the molecule and pigment additives studied here likely disrupt the packing of the polymer backbones, yielding more amorphous PLLA with better solvent/enzyme access and allowing for greater biodegradation.

## Conclusions

Our study demonstrated that total enzyme hydrolysis was heightened by environmentally relevant treatment, where UV degradation of PLA causes an increase in biodegradation. This relationship is the result of the reduced molecular weight caused by photoaging. Further, the presence of additives serves a similar purpose in altering the packing of the polymer to allow for expedited biodegradation. Ultimately, this study serves to identify the effects of environmentally relevant degradability on waste PLLA to better inform its design and disposal. As plastic pollution continues to grow, it is necessary to evaluate these assumptions in follow-up studies to evaluate, consolidate, and expand the discussed results.

## Supporting Information Available

Methods to optimize enzyme concentration, calibrating fluorescence dilaurate with fluorescence spectroscopy, tables of hydrolysis quantification values, hydrolysis curves of standards, enzyme activity assay data and discussion, table of reaction rate fits, FTIR polymer analysis, DSC thermograms and melting point analysis, and commercial PLA product enzyme hydrolysis data (PDF)
