# Supplementary material for: UV Light Degradation of Polylactic Acid Kickstarts Enzymatic Hydrolysis
Source: ACS Mater Au. 2023 Nov 2;4(1):92–8. doi: 10.1021/acsmaterialsau.3c00065 (PMC10786133; doi:10.1021/acsmaterialsau.3c00065)
Supplement: Supplementary file 1 — mg3c00065_si_001.pdf [file mg3c00065_si_001.pdf]

Supporting Information

UV Light Degradation of Polylactic Acid Kickstarts Enzymatic Hydrolysis

Margaret H. Brown, Thomas D. Badzinski, Elizabeth Pardoe, Molly Ehlebracht and Melissa A. Maurer-Jones*

Department of Chemistry and Biochemistry, University of Minnesota Duluth, 1038 University Dr, Duluth, MN 55812

*maujones@d.umn.edu

ADDITIONAL METHODS:

Optimization of Enzyme Concentration

Initial experimentation using ultraviolet-visible spectroscopy was performed to ensure optimal enzymatic hydrolysis conditions. Pristine PLLA samples were exposed to a variety of enzyme concentrations over a 4 h period. Absorbances (λ=280 nm) were taken of solutions prior to and following plastic exposure to determine the amount of proteinase K binding to the polymer surface and converted to a concentration with a calibration curve (not shown). SI Figure S1 shows the solution concentration of enzyme before exposure to polymer and the change in solution concentration 4 h incubation with PLLA. The changes in concentration of proteinase K are not statistically significantly different. This indicates there is not a concentration dependent binding of the enzyme to the plastic in these concentration ranges and likely the relationship between proteinase K and the polymer surface is dynamic. Due to these results, amid range concentration of 30 μM was used for further experimentation.

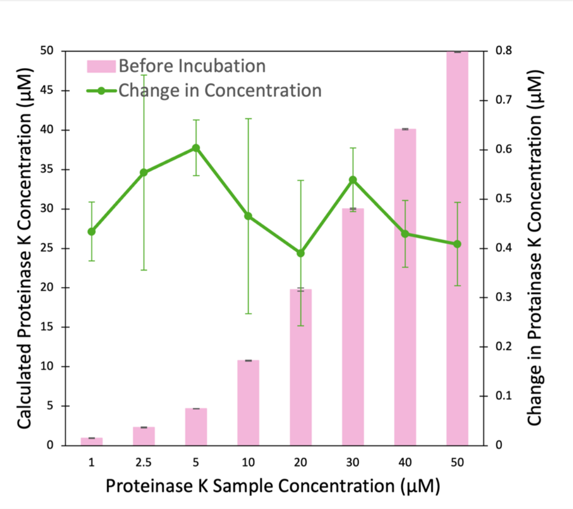

Figure S1: Comparison of the change in concentration of Proteinase K in solution when exposed to PLLA. Bars represent the calculated concentrations of initial proteinase K solution conditions by using the calibration curve data. The line represents the difference in initial and final concentrations after PLLA exposure. Values represent the average of triplicate samples with error bars representing the standard deviation.

Fluorescein Dilaurate (FDL) Calibration

To ensure proteinase K could hydrolyze FDL and cause a fluorescent signal, the same casting technique as described in the main text was used to place FDL in the bottom of a cuvette at the same amount as was embedded in the PLLA matrix. Figure S2 demonstrates the total fluorescent signals of selected time points over a 6 h period. After 55 min, a bathochromic shift in the fluorophore's spectra is observed. To determine the cause of such a shift, a calibration curve of fluorescein was developed over the range of 25 to 400 nM, shown in Figure S3. Again, this bathochromic shift is demonstrated, which begins at concentrations greater than 25 nM. Due to this shift, Figure S4 was compiled as a way to quantify fluorescein concentrations that surpass 25 nM of fluorescein (50nM ester bonds) in experiments. Concentration is plotted to peak maximum

and correlated to a logarithmic curve. This equation allows for a way to calculate ester bond breakage despite surpassing the linear range of 0-25 nM, although using the bathochromic shift as the calibration signal should be considered semi-quantitative. Due to shifting, a calibration curve of up to 25 nM was developed to reach a linear range. This is demonstrated in Figure S5 and is used to calculate the percentage of ester bonds broken when total degradation releases a concentration of fluorescein less than 25 nM. To ensure the autohydrolysis of FDL when cast in PLLA was accounted for a reference cuvette was run with each experiment. Figure S6 displays the raw fluorescent signal of a reference cuvette. Figure S7 demonstrates a percentage of polyester bonds broken over time of one of these reference cuvettes for a pristine PLLA sample. In calculations, the reference raw signal is subtracted from each individual sample prior to averaging and further manipulation.

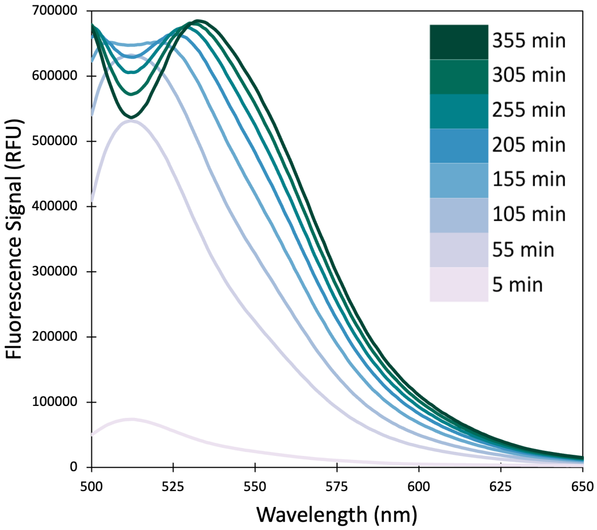

Figure S2: Fluorescent spectra of fluorescein dilaurate (FDL) hydrolyzing over time by proteinase K. Each spectra represents increased time FDL was exposed to proteinase K. Spectra represents the average of triplicate spectra. Error not shown to better visualize spectra.

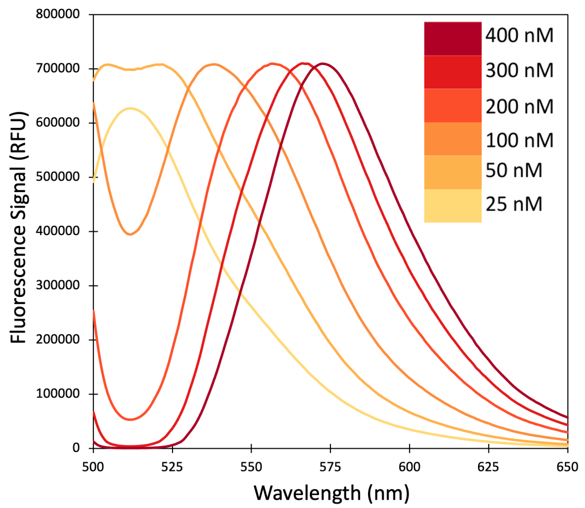

Figure S3: Fluorescent spectra of 25, 50, 100, 200, 300, and 400 nM concentrations of FDL. Spectra represents the average of triplicate spectra. Error not shown to better visualize spectra.

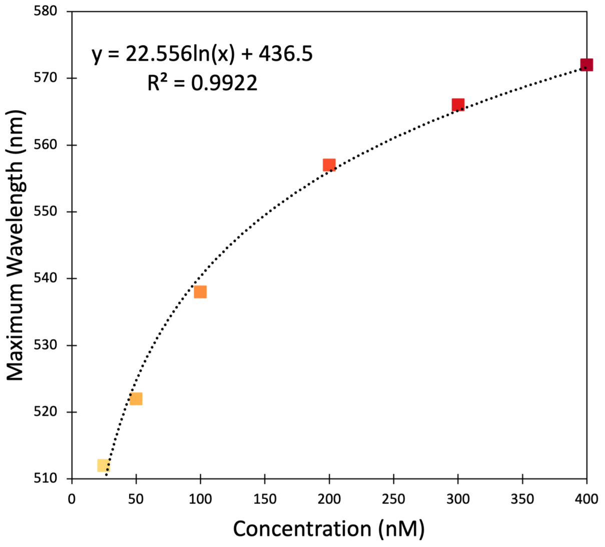

Figure S4: Wavelength peak maximum vs concentration of 25, 50, 100, 200, 300, and 400 nM concentrations of FDL. Measurements were then fit to a logarithmic scale. Markers represent the average of triplicate values with error bars, standard deviation, too small to observe.

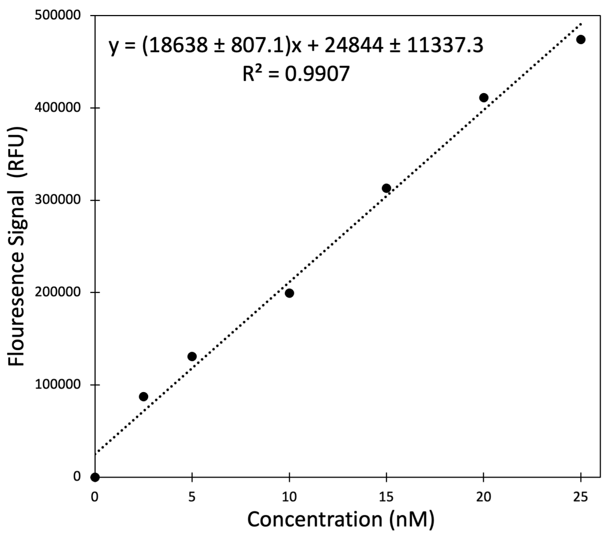

Figure S5: Calibration curve of fluorescein over 0 to 25 nM concentration range. Measurements were then fit to a line. Markers represent the average of triplicate values with error bars, standard deviation, too small to observe.

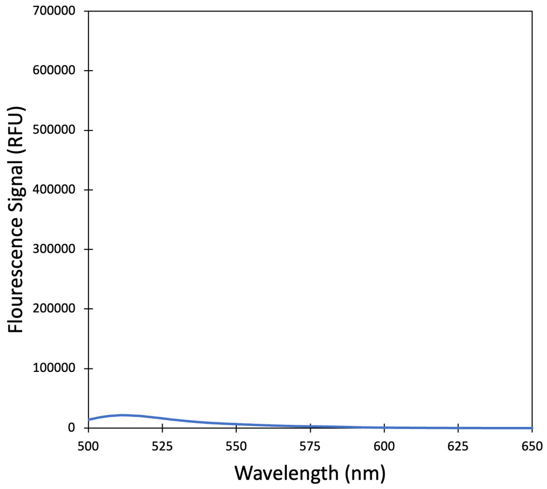

Figure S6: Fluorescent spectra of reference cuvette.

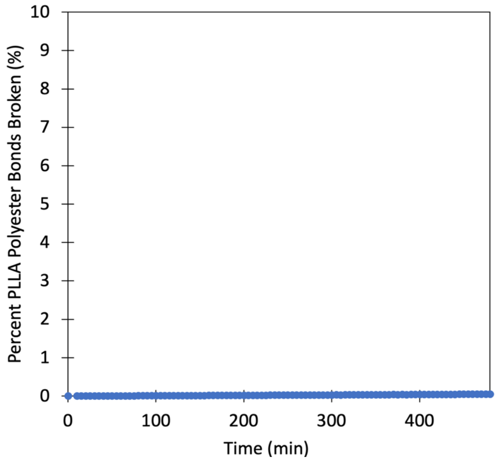

Figure S7: Example reference cuvette percentage of PLLA polyester bonds hydrolyzed by buffer over time of pristine PLLA.

ADDITIONAL RESULTS

Additional Enzymatic Hydrolysis Work

Below is additional information regarding the enzymatic hydrolysis of aged PLLA.

The total percent (%) of PLLA polyester bonds broken for each irradiation time point was compiled (Table S1). Additionally, a similar table was compiled of the same data from the experiment utilizing lipase, rather than proteinase K (Table S2).

Table S1: Observed average total percentage (± standard deviation) of polyester bonds broken (%) for pristine, 1h, 2 h, 3 h, 4 h, 8 h, and 24 h irradiated PLLA by proteinase K upon 8 h incubation.

Total Enzymatic Hydrolysis of Aged PLLA	
Irradiation Time (h)	Percent PLLA Polyester Bonds Broken (%)
0	5.4 ± 0.06
1	5.6 ± 0.07
2	7.1 ± 0.11
3	8.5 ± 0.01
4	10.3 ± 0.21
8	24.9 ± 0.00
24	79.9 ± 0.00

Table S2: Observed average total percentage (± standard deviation) of polyester bonds broken (%) for pristine, 1 h, 2 h, 3 h, and 4 h irradiated PLLA by lipase upon 8 h incubation.

Total Enzymatic Hydrolysis of Aged PLLA Utilizing Lipase	
Irradiation Time (h)	Percent PLLA Polyester Bonds Broken (%)
0	3.4 ± 0.32
1	3.8 ± 0.19
2	3.7 ± 0.33
3	8.1 ± 0.33
4	9.5 ± 0.03

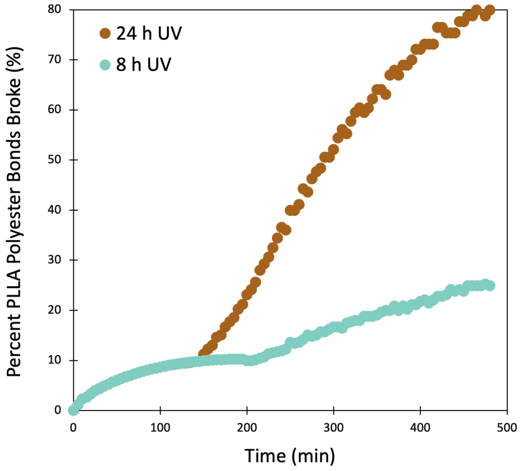

Figure S8: Percentage of PLLA polyester bonds broken over time of 8h and 24h irradiated PLLA. Markers represent the average of triplicate values with error bars, standard deviation, too small to observe.

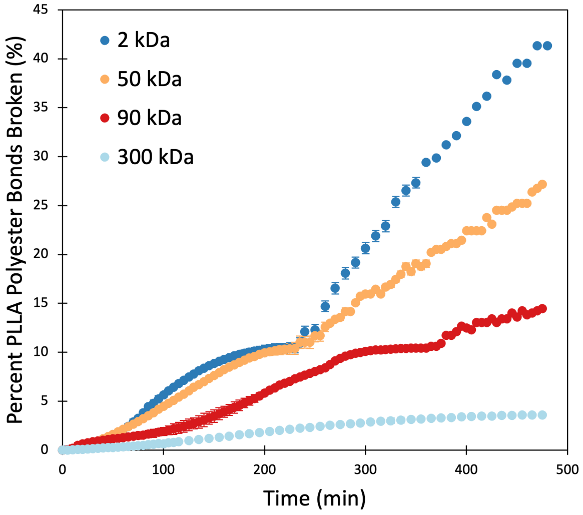

Figure S9: Percentage of PLLA polyester bonds broken over time of 2, 50, 90, and 200 kDa PLLA molecular weight standards. Markers represent the average of triplicate values with error bars the standard deviation. It should be noted that the shape of the hydrolysis curves is likely an artifact of calibration procedures, where the bathochromic shifts in the fluorophore spectrum above 25 nM has a different calibration procedure that yields more semi-quantitative results as described in SI FDL Calibration section.

Enzyme Activity

Over the course of experimentation a consistent plateau in enzymatic hydrolysis of PLLA was observed. To investigate if this plateau was a result of exhausted enzyme activity, two separate experiments were performed on aged (4 h irradiated) PLLA. After the 8 h of enzyme exposure, as done throughout experimentation, the enzyme solution was removed from the cuvette and replaced with fresh solution (Figure S10). As demonstrated with the new enzyme present, hydrolysis takes off once again. In a secondary experiment, rather than replacing exhausted enzyme after 8 h, fresh enzyme were spiked into the existing solution and measured for an additional 8 h (Figure S11). This second experiment proved less obvious in demonstrating the exhaustion of enzyme because as the 400 min mark is approached, FDL concentration surpasses 25 nM. As a result the logarithmic relationship defined in Figure S4 is used to semi-quantitatively calculate ester bond breakage.

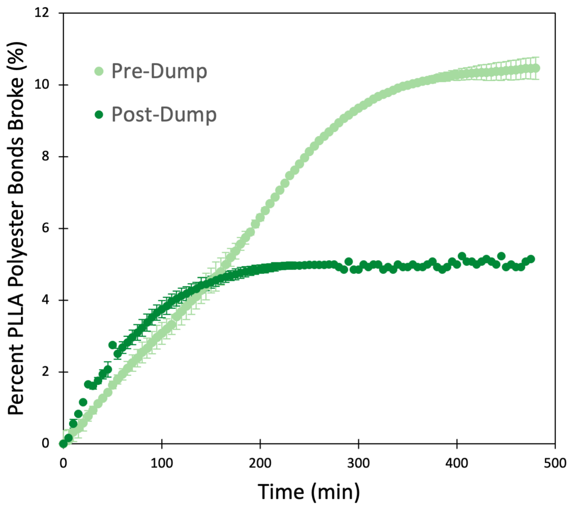

Figure 10: Percentage of 4 h irradiated PLLA polyester bonds broken over an initial 8 h period (Pre-Dump). Further depicted is an additional 8 h period (Post-Dump) in which initial enzyme solution was removed and replaced.

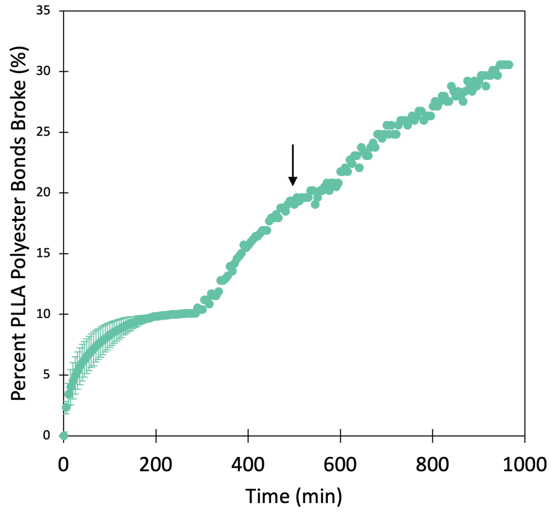

Figure S11: Percentage of 4 h irradiated PLLA polyester bonds broken with fresh enzyme spiked into existing solution after 8 h. Arrow indicates time of enzyme spike.

Table S3: Observed R^2 values for each irradiation time point after being fit to zero and first reaction rate laws.

Irradiation Time (h)		0	1	2	3	4
Zero Order Fit	Slope (k)	0.0126	0.0105	0.0135	0.1870	0.0236
	R^2 Value	0.9254	0.9389	0.9019	0.8560	0.8900
First Order Fit	Slope (k)	0.0067	0.0049	0.0049	0.0055	0.0079
	R^2 Value	0.6381	0.6828	0.5440	0.5923	0.5367

Fitting the enzyme hydrolysis data from Figure 1 to reaction rate kinetics. Reaction rate constants were determined by linearizing the first order reaction kinetic equations and fitting both the zero and first order fits to a linear trendline. Results (Table S3) revealed zero-order kinetics are the best fit (i.e., higher R^2 values), however, the change in the enzyme catalytic behavior in the above figures likely means the reaction rates are more challenging to interpret and should be considered with a large amount of error as is.

Additional Polymer Characterization

FTIR: Additional measurements achieved in polymer characterization are presented below. FTIR measurements were taken after 8 h of enzyme exposure as another means to indicate markers of degradation. Similarly to what is presented in Figure 2, carbonyl and hydroxyl indices of irradiated PLLA were calculated following enzyme exposure (Figure S12). Again, ANOVA tests revealed no statistically significant difference between irradiation time points of PLLA films. Additionally, further ANOVA testing revealed no statistically significant difference in hydroxyl and carbonyl indices, respectively, when prior and following enzyme exposure indices were compared.

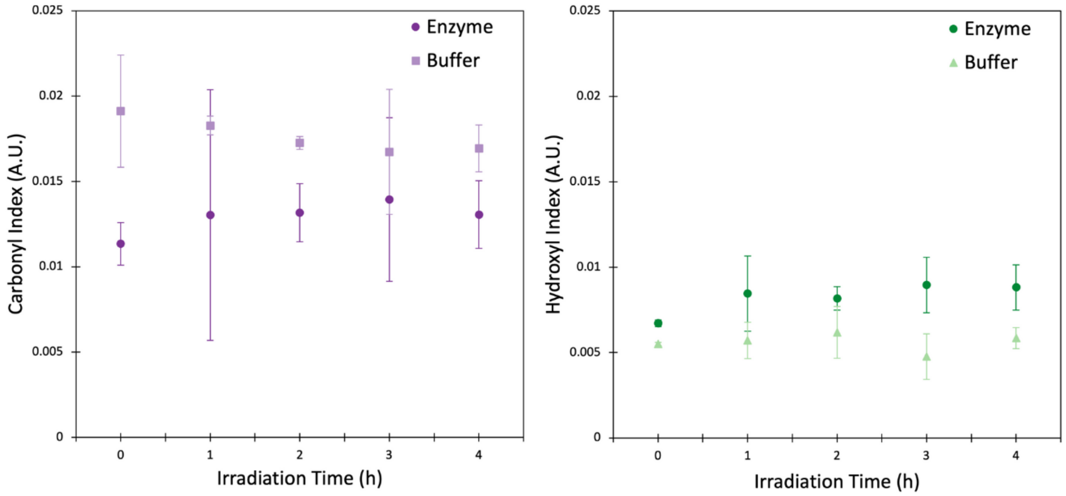

Figure S12: Carbonyl and hydroxyl indices of irradiated PLLA samples after 8 h of proteinase K exposure.

DSC: Additional data is presented below on DSC characterization. An example DSC thermogram is provided of pristine and 4 h irradiated PLLA (Figure S13). DSC measurements of PLLA

molecular weight standards were performed and utilized to confirm literature trends in regards to molecular weight and peak temperature relationships. Plotted is the average peak temperature of each molecular weight standard. A clear growth in peak temperature with increased molecular weight is observed (Figure S14). These values were also compiled into a table (Table S3).

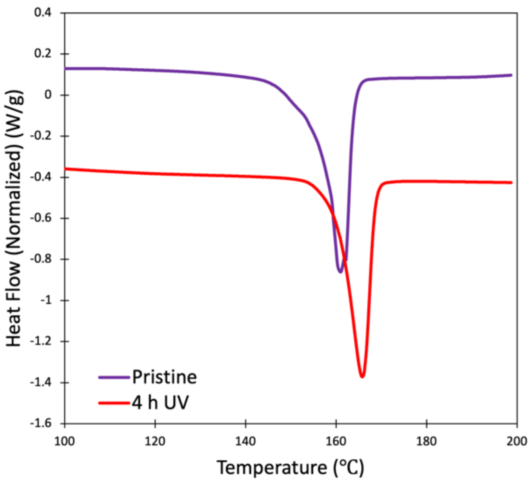

Figure S13: Example DSC thermograms of pristine and 4 h irradiated PLLA

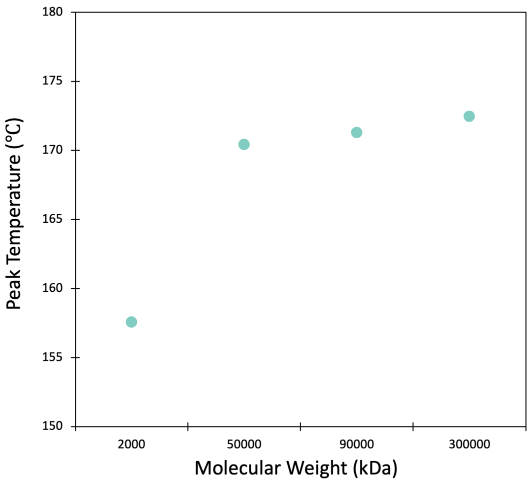

Figure S14: Observed average peak temperatures (°C) of 2, 50, 90, and 300 kDa PLLA molecular weight standards plotted. Markers represent the average of triplicate values with error bars, standard deviation, too small to observe.

Table S4: Observed average peak temperatures (°C) (± standard deviation) of 2, 50, 90, and 300 kDa PLLA molecular weight standards.

Peak Temperatures of PLLA Molecular Weight Standards	
Molecular Weight Standard (kDa)	Peak Temperature (°C)
2	157.6 ± 0.19
50	170.4 ± 0.06
90	171.3 ± 0.19
300	172.2 ± 0.32

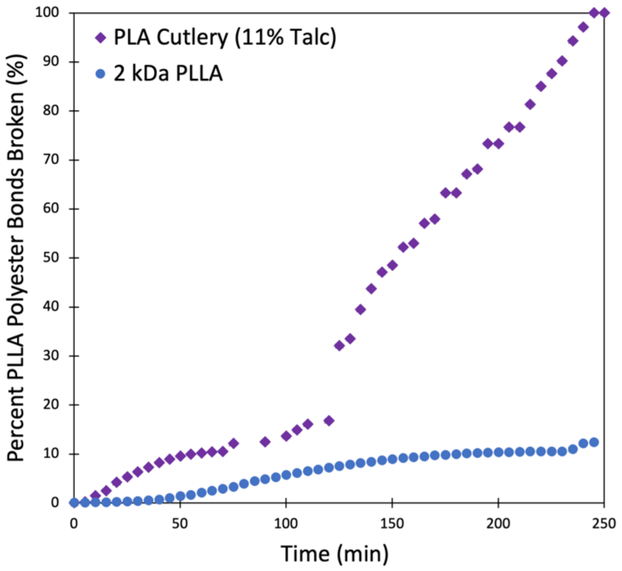

Figure S15: Percentage of PLLA polyester bonds broken over time of 2 kDa PLLA molecular weight standard and PLA cutlery comprising 11% talc. Markers represent the average of triplicate values with error bars, standard deviation, too small to observe.
